# Up-regulation of HER2 by gemcitabine enhances the antitumor effect of combined gemcitabine and trastuzumab emtansine treatment on pancreatic ductal adenocarcinoma cells

**DOI:** 10.1186/s12885-015-1772-1

**Published:** 2015-10-16

**Authors:** Shin Kan, Shigeo Koido, Masato Okamoto, Kazumi Hayashi, Masaki Ito, Yuko Kamata, Hideo Komita, Eijiro Nagasaki, Sadamu Homma

**Affiliations:** 1 Division of Oncology, Research Center for Medical Sciences, Jikei University School of Medicine, Tokyo, Japan; 2 Division of Gastroenter ology and Hepatology, Department of Internal Medicine, Jikei University School of Medicine, Tokyo, Japan; 3 Division of Oncology and Hematology, Department of Internal Medicine, Jikei University School of Medicine, Tokyo, Japan; 4 Department of Advanced Immunotherapeutics, Kitasato University School of Pharmacy, Tokyo, Japan; 5 Shimbashi Medical Checkup Office, Jikei University Hospital, Tokyo, Japan

**Keywords:** Pancreatic ductal adenocarcinoma, Human epidermal growth factor receptor 2, Gemcitabine, Trastuzumab emtansine, Combination therapy

## Abstract

**Background:**

Although pancreatic ductal adenocarcinomas (PDAs) widely express HER2, the expression level is generally low. If HER2 expression in PDA cells could be enhanced by treatment with a given agent, then combination therapy with that agent and trastuzumab emtansine (T-DM1), a chemotherapeutic agent that is a conjugate of trastuzumab, might lead to significant antitumor effects against PDA.

**Methods:**

Cell proliferation was examined by spectrophotometry. HER2 expression was examined by flow cytometry, immunoblot and quantitative reverse transcription polymerase chain reaction. T-DM1 binding to cells was examined by flow cytometry and enzyme-linked immunosorbent assay.

**Results:**

Out of 5 tested human PDA cell lines, including MIA PaCa-2, three showed increases in HER2 expression after gemcitabine (GEM) treatment. The binding of T-DM1 to GEM-treated MIA PaCa-2 cells was higher than to untreated MIA PaCa-2 cells. Treatment with GEM and T-DM1 showed synergic cytotoxic effects on MIA PaCa-2 cells *in vitro*. Cells in the G2M phase of the cell cycle were retained after GEM treatment and showed higher levels of HER2 expression, possibly contributing to the synergic effect of GEM and T-DM1.

**Conclusions:**

Combined treatment with GEM and T-DM1 might confer a potent therapeutic modality against PDA as a result of GEM-mediated HER2 up-regulation.

**Electronic supplementary material:**

The online version of this article (doi:10.1186/s12885-015-1772-1) contains supplementary material, which is available to authorized users.

## Background

Pancreatic ductal adenocarcinoma (PDA) is presently the fifth leading cause of cancer-related deaths in Japan [1]. As PDA shows highly infiltrative and metastatic behavior and because most PDA patients are at an advanced stage upon diagnosis, the number of PDA patients who qualify for curable surgical treatment is low. Furthermore, even if PDA is resected, it exhibits frequent recurrence after surgical treatment, and its 5-year survival rate is only 18.8 % [2]. Accordingly, the establishment of an effective therapeutic modality is an urgent issue.

Human epidermal growth factor receptor 2 (HER2) is a 185-kDa transmembrane glycoprotein with tyrosine kinase receptor activity, which provides signal transduction for cell proliferation and differentiation [3]. It has been reported that overexpression of HER2 is found in 15-25 % of breast cancers and is related to poor prognosis [4, 5]. Trastuzumab, a monoclonal antibody that recognizes the HER2 protein, specifically binds to the extracellular HER2 receptor and elicits anti-tumor activity by blocking signal transduction and antibody-dependent cell-mediated cytotoxicity (ADCC) [6]. The treatment of HER2-overexpressing breast or gastric cancer patients with trastuzumab in combination with chemotherapeutic agents is being evaluated in phase III clinical studies and is more effective than standard chemotherapy [7, 8]. It is known that PDAs widely express HER2 (10-82 %) [9–13]. Although trastuzumab treatment against human PDA cells has shown significant antitumor activity in basic *in vivo* and *in vitro* studies, the combined treatment of gemcitabine (GEM) or capecitabine with trastuzumab against metastatic PDA did not result in improved progression-free survival or overall survival, possibly because the expression and gene amplification of HER2 in PDAs were generally lower than in breast cancer [14, 15].

Trastuzumab emtansine (T-DM1) is a recently developed conjugate of trastuzumab and DM1 (derivative of maytansine 1), a chemotherapeutic agent. When T-DM1 binds to cell-surface HER2 receptors, it is delivered into the lysosome via endocytosis and digested. The active form of DM1 is released into the cell and inhibits the assembly of microtubules [16, 17]. Therefore, T-DM1 exerts selective anti-tumor effects more strongly than trastuzumab. In preclinical studies, the cytotoxic activity of T-DM1 in breast and gastric cancer cells was stronger than that of trastuzumab, even if the tumor cells were resistant to trastuzumab [18, 19]. In clinical studies, the antitumor effect of T-DM1 was found to be superior to those of lapatinib and docetaxel in patients with HER2-positive advanced breast cancer that was resistant to trastuzumab and taxanes [20].

One possible reason why trastuzumab has not been applied as a treatment for PDA might be because PDA cells exhibit low levels of HER2 expression. Therefore, if HER2 expression in PDA cells could be enhanced by treatment with some agent, combination therapy with that agent and T-DM1 might show a significant antitumor effect because more T-DM1 could be delivered into PDA cells. We have found that GEM can enhance HER2 expression in PDA cells; as such, a combined treatment of GEM and T-DM1 may provide a potent therapeutic effect against PDA. In the present study, HER2 up-regulation by GEM treatment and the synergistic cytotoxic effect of GEM and T-DM1 against PDA cells were examined.

## Methods

### Cell lines and agents

The human pancreatic adenocarcinoma (PDA) cell lines MIA PaCa-2, PANC-1, AsPC-1, Capan-1 and Capan-2 were obtained from the American Type Culture Collection (Manassas, VA, USA) [21]. All cell lines were cultured in Dulbecco’s modified Eagle medium (Nissui Pharmaceutical, Tokyo, Japan) supplemented with penicillin/streptomycin (Life Technologies, Carlsbad, CA, USA) and 10 % heat-deactivated fetal bovine serum. Gemcitabine (GEM) was purchased from Eli Lilly Japan (Kobe, Japan), five-fluorouracil (5FU) was purchased from Kyowa Hakko Kirin Co. (Tokyo, Japan), and oxaliplatin (L-OHP) was purchased from Sigma-Aldrich (St. Louis, MO, USA). Trastuzumab was a gift from Chugai, Inc. (Tokyo, Japan), and trastuzumab emtansine (T-DM1) was provided by Genentech Inc. (South San Francisco, CA, USA).

### Flow cytometric analysis

To assess HER2 expression levels, cells were incubated either with phycoerythrin (PE)-labeled anti-human HER2 (24D2) or with corresponding isotype-control antibodies (BioLegend, San Diego, CA, USA) in buffer (1 % FBS, 2 mM EDTA and 0.1 % NaN_3_ in PBS) for 30 min at 4 °C, after which they were washed and then analyzed using a MACSQuant Analyzer (Miltenyi Biotech K.K., Bergisch Gladbach, Germany). Before using the analyzer, 4 μg/ml propidium iodide (PI) (Sigma-Aldrich, St. Louis, MO, USA) was added to each sample to exclude dead cells.

To assess T-DM1 binding to PDA cells, 0.5x10^6^ cells were incubated with 30 μg/ml T-DM1 at 37 °C for 1 h. The cells were washed, incubated with PE-labeled anti-human IgG Fc (HP6017) (Biolegend) or corresponding isotype-control antibodies (Affymetrix, Santa Clara, CA, USA) in buffer for 30 min at 4 °C, washed, re-suspended and analyzed using a MACSQuant Analyzer (Miltenyi Biotech K.K.). Before using the analyzer, 4 μg/ml PI (Sigma-Aldrich) was added to the sample to exclude dead cells. The mean fluorescence intensity (MFI) of HER2 was analyzed using MACSQuantify Software.

### Cell cycle analysis

MIA PaCa-2 cells were suspended in Hoechst 33342 (5 μg/ml) (Life Technologies) and incubated at 37 °C for 90 min, then washed and incubated with phycoerythrin (PE)-labeled anti-human HER2 (24D2) or the corresponding isotype control antibodies (BioLegend) for 30 min at 4 °C. Before using the analyzer, 1 μg/ml PI (Sigma-Aldrich) was added to the sample to exclude dead cells. In cell cycle analysis, the mean fluorescence intensity (MFI) of HER2 in each phase of the cell cycle was examined using MACSQuant Analyzer (Miltenyi Biotech K.K.) and MACSQuantify Software.

### Quantitative reverse transcription polymerase chain reaction (qRT-PCR)

The cells were lysed in RLT Plus Buffer (Qiagen, Hilden, Germany) and homogenized. From 2 μg of total RNA, cDNA was synthesized using a High-Capacity cDNA Reverse Transcription kit (Applied Biosystems, Foster City, CA, USA) and a GeneAmp PCR System 9700 (Applied Biosystems). For qRT-PCR detection of HER2 and 18S rRNA, 5 ng of cDNA was amplified using SYBR Premix Ex Taq II (Takara Bio Inc., Otsu, Shiga, Japan) and a 7300 Real-Time PCR System (Applied Biosystems). The PCR conditions consisted of an initial denaturation step (95 °C for 30 s), followed by 40 cycles (95 °C for 5 s and 62 °C for 31 s) and a dissociation step (95 °C for 15 s, 60 °C for 60 s and 95 °C for 15 s, and 60 °C for 15 s). The sequences of the primers (Operon Biotechnologies K.K., Tokyo, Japan) for HER2 and the 18S ribosomal RNA (rRNA) that were used in the present study were as follows: 5’-TCCTGTGTGGACCTGGAT-3’ as a forward primer and 5’-TGCCGTCGCTTGATGAG-3’ as a reverse primer for human HER2; 5’-CGGCTACCACATCCAAGGAA-3’ as a forward primer and 5’-GCTGGAATTACCGCGGCT-3’ as a reverse primer for human 18S rRNA. The data were analyzed using the comparative ΔΔCT method by calculating the difference between the threshold cycle (CT) values of the target and reference genes of each sample and then comparing the ΔCT values of each drug treatment to the non-treated group.

### Immunoblot analysis

Cells were homogenized in 20 mM HEPES buffer containing 1 % Triton X-100, 100 mM PMSF, 1 mg/ml leupeptin, 1 mg/ml aprotinin and 0.9 M Na_3_VO_4_, and the protein concentrations of the homogenates were analyzed using a Pierce BCA Protein Assay Kit (Thermo Fisher Scientific Inc., Waltham, MA, USA). Protein samples (10 μg) were separated by electrophoresis on a 7.5 % sodium dodecyl sulfate-polyacrylamide gel (ATTO, Tokyo, Japan) and transferred to a polyvinylidene difluoride membrane (Bio-Rad Laboratories, Hercules, CA, USA). After blocking with 3 nonfat milk and 3 % bovine serum albumin for 1 h, the membrane was treated with Abs against HER2 (1:1000) (e2-4001 + 3B5, Thermo Fisher Scientific Inc., Waltham, MA, USA) and glyceraldehyde 3-phosphate dehydrogenase (GAPDH) (1:600,000) (2D4A7, Abcam Inc, Cambridge, UK) and then with horseradish peroxidase-conjugated secondary antibodies (Cell Signaling Technology Inc., Danvers, MA, USA). Chemi-Lumi One Super (Nakalai Tesque Inc, Kyoto, Japan) was used for chemiluminescent detection.

When HER2 was detected by immunoblot analysis, it produced two bands of approximately 185 and 155 kDa. The 155 kDa band has been reported to represent cytoplasmic HER2, whereas the 185 kDa represents membrane-bound HER2 [22–24].

### Enzyme-linked immunosorbent assay (ELISA)

GEM- and T-DM1-treated MIA PaCa-2 cells (1x10^6^ cells) were homogenized in 20 mM HEPES buffer containing 1 % Triton X-100, 100 mM PMSF, 1 mg/ml leupeptin, 1 mg/ml aprotinin and 0.9 M Na_3_VO_4_. To quantify human IgG (T-DM1) binding to 1x10^6^ MIA PaCa-2 cells, a human IgG total Ready-Set-Go kit (Affymetrix, Santa Clara, CA, USA) was used according to the manufacturer’s procedures.

### Estimation of the anti-proliferative effects of the agents by an *in vitro* cell growth assay

**Fig. 1:**
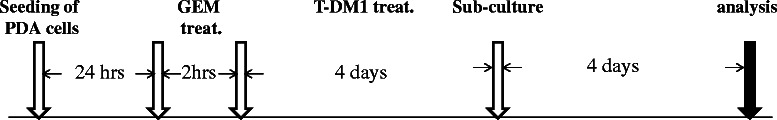
A schematic illustrating how the treatments were performed on MIA PaCa-2 and Capan-1 cells

MIA PaCa-2 cells and Capan-1 cells were treated with GEM (0, 30, 100 or 300 ng/ml) for 2 h. After washing the cells with PBS, they were incubated in medium containing T-DM1 (0, 10 or 30 μg/ml) for 96 h. Then, the cells were detached by trypsin-EDTA treatment, and the number of cells that were not stained with trypan blue was determined using a hemocytometer. Identical numbers of viable cells were seeded into 96-well plates (10^3^/100 μl/well). After a 96-h incubation, cell growth was examined by spectrophotometry using the counting reagent SF (Nakarai Tesque Inc, Catalog No. 07553). The cell count reagent SF is a sensitive colorimetric reagent that utilizes tetrazolium salt, which is highly water-soluble. Because the absorbance at 450 nm is proportional to the number of viable cells in the medium, the viable cell number can be determined using the absorbance value of a previously prepared calibration curve. Fluorescence was measured at 450 nm using an iMark microplate absorbance reader (Bio-Rad Laboratories, Hercules, CA, USA). The treatment design is shown in Fig. 1.

### Statistical analysis

All data are presented as the mean ± standard deviation (SD). Comparisons between the untreated control and drug-treated groups were performed by non-paired Student’s t-tests for two independent groups and with Dunnett’s method for multiple-group comparisons. A p-value of <0.05 was considered statistically significant. Statistical analyses were performed using Microsoft Office Excel 2007 (Microsoft Corporation, Redmond, WA, USA) with the add-on software Statcel3 (OMS publishing Inc., Saitama, Japan).

## Results

### GEM treatment increased HER2 expression in PDA cells with originally low or moderate HER2 expression

**Fig. 2:**
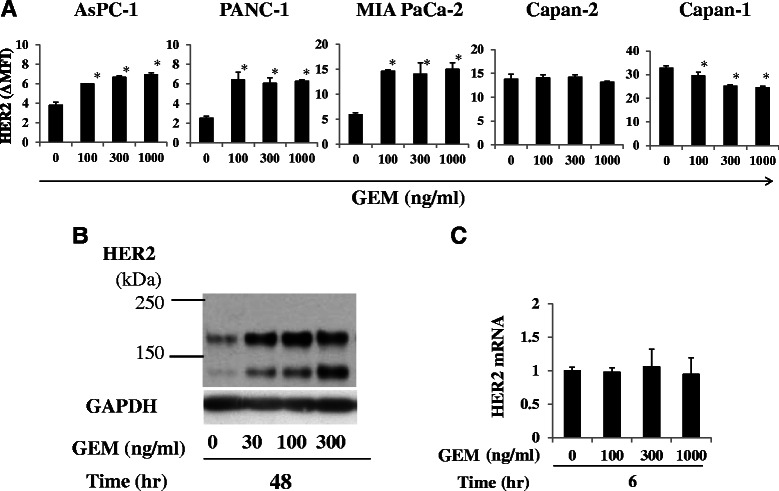
HER2 expression was up-regulated by GEM treatment in some PDA cell lines. **a** Five PDA cell lines were either untreated or treated with GEM for 2 h, washed with PBS and incubated for another 48 h in fresh medium. HER2 expression was analyzed by flow cytometry. HER2 expression (ΔMFI) was calculated as the MFI of HER2 minus that of an isotype control. *P < 0.01 (*n* = 3) vs. untreated samples. **b** GEM-treated MIA PaCa-2 cells (0, 30, 100 and 300 ng/ml, 2 h) were incubated for 48 h, and the expression of the HER2 protein in the whole-cell lysates was analyzed using immunoblots. GAPDH was used as an internal control. **c** GEM-treated MIA PaCa-2 cells (0, 100, 300 and 1000 ng/ml, 2 h) were incubated for 2, 6, 12, 24 and 48 h, and the HER2 mRNA level in each treated cell line was determined using qRT-PCR. The HER2 mRNA expression levels were normalized to that of 18S ribosomal RNA and quantified using the ΔΔCt method. The level of HER2 mRNA at 6 h after GEM treatment is shown as a representative

PANC-1, AsPC-1 and MIA PaCa-2 cells showed significant increases in HER2 expression after GEM treatment, but HER2 expression in Capan-2 cells was not affected (Fig. 2a). On the contrary, GEM treatment suppressed HER2 expression in Capan-1 cells. PANC-1, AsPC-1 and MIA PaCa-2 cells, which showed up-regulated HER2 expression after GEM treatment, originally showed low or moderate HER2 expression levels. Conversely, Capan-2 and Capan-1 cells, which did not show HER2 up-regulation after GEM treatment, exhibited considerably high levels of HER2 expression prior to treatment (Fig. 2a).

The augmentation of HER2 expression in MIA PaCa-2 cells following GEM treatment was also examined by immunoblot analysis. As shown in Fig. 2b, HER2 expression levels, of both membranous and cytoplasmic forms, in whole cells was enhanced by GEM treatment. However, the expression of HER2 mRNA was not enhanced 2, 6, 12, 24 or 48 h after GEM treatment (Fig. 2c, Additional file 1: Figure S1).

### The effects on HER2 expression levels of various standard chemotherapeutic agents for PDA treatment

**Fig. 3:**
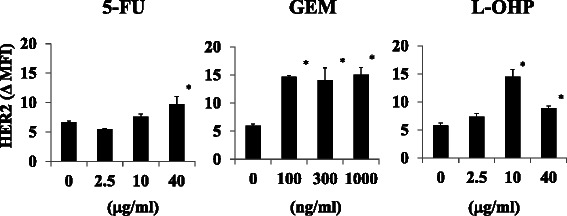
Augmentation of HER2 expression in MIA PaCa-2 cells using standard drugs for PDA treatment. MIA PaCa-2 cells were treated with 5-FU (0, 2.5, 10 or 40 μg/ml) for 1.5 h, GEM (0, 100, 300 or 1000 ng/ml) for 2 h or L-OHP (0, 2.5, 10 or 40 μg/ml) for 3 h, and HER2 expression was examined 48 h after each treatment by flow cytometry. ΔMFI of HER2 was calculated as the MFI of HER2 minus that of the isotype control. *P < 0.01 vs. untreated samples (*n* = 3)

Standard chemotherapeutic agents for PDA treatment were examined for their abilities to augment HER2 expression in MIA PaCa-2 cells. The incubation time of each agent was determined based on the time it took to disappear from the blood when used in cancer chemotherapy. Treatment with 40 μg/ml of five-fluorouracil (5-FU) enhanced cell surface HER2 expression by approximately 1.5-fold, and 10 μg/ml of oxaliplatin (L-OHP) increased HER2 expression by approximately 2.5-fold (Fig. 3). Treatment with a 100–1000 ng/ml concentration of GEM enhanced cell surface HER2 expression by approximately 2.5-fold (Fig. 3).

### Treating MIA PaCa-2 cells with GEM enhanced T-DM1 binding

**Fig. 4:**
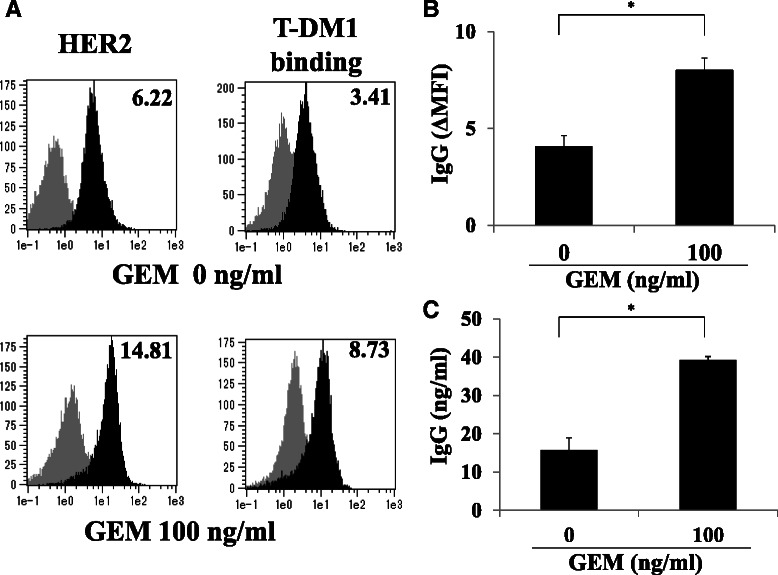
GEM treatment increased HER2 expression and resulted in increased T-DM1 binding to MIA PaCa-2 cells. **a** MIA PaCa-2 cells were either untreated or treated with GEM (100 ng/ml) for 2 h, washed with PBS and incubated in fresh medium for 48 h. The cells were detached by trypsin-EDTA treatment, untreated or treated with T-DM1 (30 μg/ml) for 1 h and then analyzed by flow cytometry using PE-labeled anti-human IgG. Left: HER2 expression in untreated or GEM-treated MIA PaCa-2 cells. Right: T-DM1 binding to untreated or GEM-treated MIA PaCa-2 cells. The grey and black areas indicate the isotype control and the specific antibody, respectively. The values on the left indicate ΔMFI for HER2, representing the MFI for HER2 minus that of the isotype control. The values on the right indicate ΔMFI for human IgG, representing the MFI for PE-labeled anti-human IgG minus that of the isotype control. **b** Untreated or GEM-treated MIA PaCa-2 cells were incubated with T-DM1 as described in (**a**), and T-DM1 binding was examined by flow cytometry using PE-labeled anti-human IgG. The ΔMFI of human IgG was calculated as the MFI of PE-labeled anti-human IgG minus that of the isotype control. *P < 0.01 (*n* = 3). **c** Untreated or GEM-treated MIA PaCa-2 cells were incubated with T-DM1 as described in (**a**). The cells (1x10^6^) were lysed, and the amount of human IgG binding to the cells was determined by ELISA. *P < 0.01 (*n* = 3)

Because treating MIA PaCa-2 cells with GEM enhanced the cell surface expression of HER2, we next examined whether T-DM1 exhibited enhanced binding to HER2 in GEM-treated MIA PaCa-2 cells. There was approximately 2-fold higher binding of T-DM1 to GEM-treated MIA PaCa-2 cells than to untreated MIA PaCa-2 cells (Fig. 4a, b). The extent of increase in T-DM1 binding to the GEM-treated MIA PaCa-2 cells paralleled the enhancement in HER2 expression following GEM treatment (Fig. 4a). The amount of T-DM1 that bound to 10^6^ MIA PaCa-2 cells, which were treated or not treated with GEM, was quantified by ELISA. There was approximately 2.5-fold greater binding of T-DM1 to GEM-treated cells than to untreated cells (Fig. 4c).

### Combined treatment with GEM and T-DM1 synergistically inhibited cell proliferation in MIA PaCa-2 cells

**Fig. 5:**
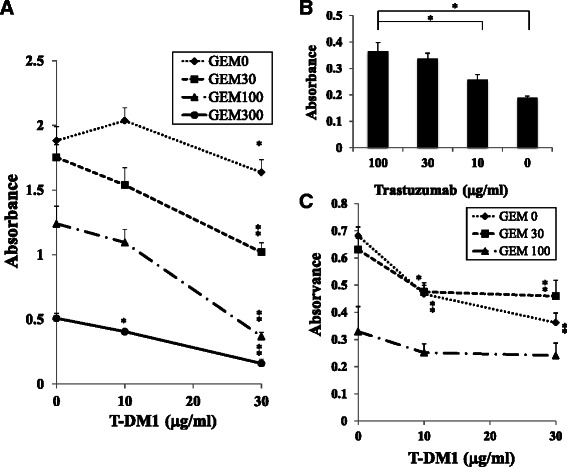
Co-treatment with GEM and T-DM1 synergistically inhibited the proliferation of MIA PaCa-2 cells. **a** MIA PaCa-2 cells were treated with GEM (0, 30, 100 or 300 ng/ml) for 2 h, washed with PBS and incubated in medium containing T-DM1 (0, 10 or 30 μg/ml) for 96 h. Then, the cells were detached by trypsin-EDTA treatment, and identical numbers of cells that were not stained with trypan blue were seeded into 96-well plates and incubated for 96 h. Cell growth was examined by spectrophotometry. *P < 0.05, **P < 0.01 vs. samples untreated with T-DM1 at each GEM concentration. The same experiment was performed twice, and similar results were obtained. **b** GEM-treated (100 ng/ml) MIA PaCa-2 cells were incubated with trastuzumab (0, 10, 30 or 100 μg/ml) in the presence of T-DM1 (30 μg/ml) for 96 h, and identical numbers of cells not stained with trypan blue were seeded into 96-well plates. After a 96-h incubation, cell growth was examined by spectrophotometry using the counting reagent SF. *P < 0.01 vs. samples treated with GEM, T-DM1 and 100 μg/ml of Trastuzumab. **c** Capan-1 cells were treated in the same way as MIA PaCa-2 cells, as described in (**a**). Cell growth was examined by spectrophotometry. *P < 0.05, **P < 0.01 vs. samples not treated with T-DM1 at each GEM concentration

Treatment with both GEM and T-DM1 showed a synergistic anti-proliferative effect when MIA PaCa-2 cells that were pre-treated with 30 or 100 ng/ml of GEM were further treated with various doses of T-DM1 (Fig. 5a). The cytotoxic activity of T-DM1 (30 μg/ml) on GEM -pretreated MIA PaCa-2 cells (100 ng/ml) was competitively inhibited when trastuzumab was added to the culture (Fig. 5b). On the contrary, a synergistic anti-proliferative effect was not observed when Capan-1 cells, which did not upregulate HER2 expression in response to GEM treatment, were treated with GEM and T-DM1 in a similar manner as MIA PaCa-2 cells (Fig. 5c).

### G2M cell population of GEM-treated MIA PaCa-2 cells showed higher HER2 expression than G1 or S cell population

**Fig. 6:**
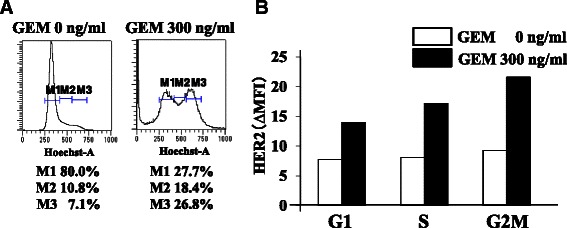
GEM-treated MIA PaCa-2 cells in G2M showed higher HER2 expression than those in S or G1. **a** MIA PaCa-2 cells were treated with GEM (0 or 300 ng/ml) for 2 h, washed with PBS and incubated for 96 h. The cells were examined for cell cycle status by flow cytometry. M1: G1 phase, M2: S phase, M3: G2M phase. **b** HER2 expression in G1, S and G2M cell populations of untreated or GEM-treated MIA PaCa-2 cells. The cell population in each phase was gated, and HER2 expression was examined by flow cytometry. HER2 (ΔMFI) was calculated as the MFI of PE-labeled anti-human HER2 minus that of the isotype control. The experiments were repeated three times with similar results, and a representative result is shown

GEM treatment of MIA PaCa-2 cells induced a marked decrease in the G1 cell population, but the G2M cell population was retained (Fig. 6a). Untreated and GEM-treated cells in each phase of the cell cycle were examined for HER2 expression. In each of the phases, HER2 expression in GEM-treated cells was higher than in untreated cells, and the G2M cell population of the GEM-treated cells exhibited higher HER2 expression than the G1 or S cell populations (Fig. 6b), while HER2 expression in untreated MIA PaCa-2 cells was virtually the same during each phase of the cell cycle.

## Discussion

In the present study, we demonstrated that the expression of HER2 in several human PDA cell lines, including in MIA PaCa-2, was enhanced by short-term treatment with GEM. GEM is a nucleoside analog that is widely used as a standard therapeutic agent against PDA [25]. Although the amount of HER2 protein increased following GEM treatment, HER2 mRNA was unaffected. It is possible that the enhanced HER2 expression following GEM treatment was promoted at the translational level but not at the transcriptional level. Micro RNAs are small, non-coding RNAs that regulate protein translation from mRNA. It has been reported that a micro RNA regulates HER2 expression [26, 27]. In breast cancer, miR-205 down regulates HER2 [27]. If GEM suppresses miR-205 function in MIA PaCa-2 cells, HER2 protein translation might be enhanced, leading to the up-regulation of the HER2 protein. It might also be conceivable that the degradation of the HER2 protein is inhibited by GEM treatment. It has been reported that the degradation of the HER2 protein is processed through poly-ubiquitination, internalization into cells and lysosomal degradation [28, 29]. If these processes are inhibited by GEM treatment, then HER2 proteins on the cell surface and in the cytoplasm might be retained longer and might therefore be present in higher numbers because of the limited degradation.

T-DM1, a conjugate of trastuzumab and emtansine, has shown promising results in breast cancer therapy [20]. It has been reported that the cytotoxic activity of T-DM1 is dependent on the extent of HER2 expression and the resulting binding of T-DM1 to HER2 in breast and gastric cancer cells [18, 19].

Induction of HER2 up-regulation in PDA cells by treatment with a chemical agent should enhance T-DM1 binding to the PDA cells, thus providing a higher cytotoxic effect against them. Of note, T-DM1 binding to GEM-treated MIA PaCa-2 cells was significantly increased by GEM-induced HER2 up-regulation. It is likely that as more T-DM1 binds to up-regulated HER2, more MIA PaCa-2 cells are damaged by T-DM1. In fact, we demonstrated that combined GEM and T-DM1 treatment led to a synergistic cytotoxic effect against MIA PaCa-2 cells, and this effect was inhibited by competitive trastuzumab treatment. Conversely, using a combined treatment of GEM and T-DM1 against Capan-1 cells did not show a synergistic effect, possibly because HER2 expression was not up-regulated in Capan-1 cells by GEM treatment. Accordingly, to obtain a synergistic anti-tumor effect by the combined treatment of GEM and T-DM1, the following factors might be important: (1) PDA cells must express HER2 even at a low level, (2) HER2 expression is up-regulated by GEM treatment.

As GEM is an inhibitor of DNA synthesis, G1 and early S cell populations might be efficiently killed following GEM treatment, whereas a G2M cell population might survive. Analysis of HER2 expression in GEM-treated MIA PaCa-2 cells in G1, S and G2M phase indicated that the G2M cell population exhibited high HER2 expression, suggesting that more T-DM1 would bind to the GEM-treated G2M cell population. This mechanism might contribute to the synergistic cytotoxic effect of GEM and T-DM1.

Based on the above results, when combining GEM and T-DM1 treatment against PDA in future clinical settings, the quantification of HER2 expression in PDA tumor tissue should be required. However, obtaining PDA tissue by biopsy from patients with advanced PDA is difficult. Alternatively, combined GEM and T-DM1 treatment might be a promising modality for patients with cancerous peritonitis caused by advanced PDA. The prognosis of PDA patients with cancerous peritonitis is extremely poor, and no effective treatment is currently superior to GEM [30–32]. The procedure for treatment against cancerous peritonitis might be as follows. First, HER2 expression in PDA cells in ascitic fluid would be determined by a proper method, such as flow cytometry. Second, HER2 up-regulation in PDA cells after systemic GEM treatment would be confirmed by examination of PDA cells from ascitic fluid. Third, intra-peritoneal injection with T-DM1 would be performed after verifying HER2 up-regulation in PDA cells. In this way, combined GEM and T-DM1 treatment might help improve the prognosis of cancerous peritonitis caused by advanced PDA.

## Conclusions

In this study, we demonstrated that T-DM1 binding to HER2 increased in GEM-treated MIA PaCa-2 cells, as GEM enhanced their levels of HER2 expression. Furthermore, a synergic anti-tumor effect was observed when the cells were co-treated with GEM and T-DM1. Combination therapy using GEM and T-DM1 represents a novel therapeutic strategy against PDA that works through GEM-mediated up-regulation of HER2 expression.

## Additional file

Additional file 1: Figure S1. HER2 mRNA levels of GEM-treated MIA PaCa-2 cells did not increase at each time point. GEM-treated MIA PaCa-2 cells (0, 100, 300 and 1000 ng/ml, 2 h) were incubated for 2, 6, 12, 24 and 48 h, and their HER2 mRNA levels were determined using qRT-PCR. HER2 mRNA expression levels were normalized to that of 18S ribosomal RNA and quantified using the ΔΔCt method. (PPTX 83 kb)

## Abbreviations

5-FU: 5-fluorouracil
GAPDH: Glyceraldehydes 3-phosphate dehydrogenase
GEM: Gemcitabine
HER2: Human epidermal growth factor receptor 2
L-OHP: Oxaliplatin
MFI: Mean fluorescent intensity
PDA: Pancreatic ductal adenocarcinoma
PE: Phycoerythrin
PI: Propidium iodide
T-DM1: Trastuzumab emtansine
