# Supplementary material for: Up-regulation of HER2 by gemcitabine enhances the antitumor effect of combined gemcitabine and trastuzumab emtansine treatment on pancreatic ductal adenocarcinoma cells
Source: BMC Cancer. 2015 Oct 16;15:726. doi: 10.1186/s12885-015-1772-1 (PMC4609140; doi:10.1186/s12885-015-1772-1)
Supplement: Additional file 1: Figure S1. — HER2 mRNA levels of GEM-treated MIA PaCa-2 cells did not increase at each time point. GEM-treated MIA PaCa-2 cells (0, 100, 300 and 1000 ng/ml, 2 h) were incubated for 2, 6, 12, 24 and 48 h, and their HER2 mRNA levels were determined using qRT-PCR. HER2 mRNA expression levels were normalized to that of 18S ribosomal RNA and quantified using the ΔΔCt method. (PPTX 83 kb) [file 12885_2015_1772_MOESM1_ESM.pptx]

## Slide 1
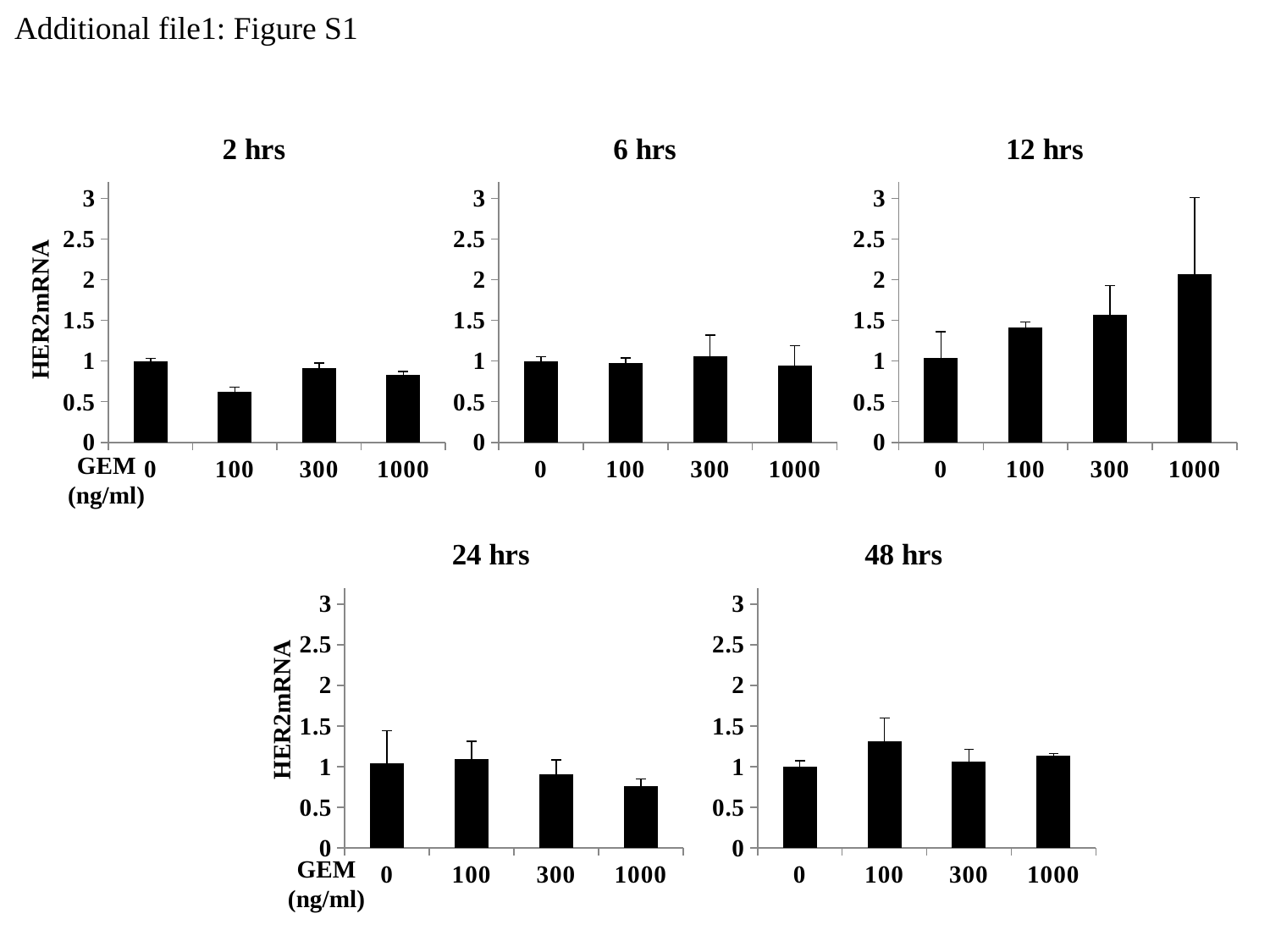

Additional file1: Figure S1
### Chart: 2 hrs
| Category | |
|---|---|
| 0.0 | 1.00034047952301 |
| 100.0 | 0.620564884550014 |
| 300.0 | 0.917925062640717 |
| 1000.0 | 0.827165802198891 |
### Chart: 6 hrs
| Category | |
|---|---|
| 0.0 | 1.001153424909512 |
| 100.0 | 0.974368383541194 |
| 300.0 | 1.059082448082923 |
| 1000.0 | 0.943929380818388 |
### Chart: 12 hrs
| Category | |
|---|---|
| 0.0 | 1.0331538676899 |
| 100.0 | 1.412117550263748 |
| 300.0 | 1.572530629814484 |
| 1000.0 | 2.070673762657427 |HER2mRNA
GEM
(ng/ml)
### Chart: 24 hrs
| Category | 24h |
|---|---|
| 0.0 | 1.044810298345958 |
| 100.0 | 1.091243947017541 |
| 300.0 | 0.903754759268005 |
| 1000.0 | 0.754595870336465 |
### Chart: 48 hrs
| Category | |
|---|---|
| 0.0 | 1.001476228251512 |
| 100.0 | 1.314505686894309 |
| 300.0 | 1.058958302275295 |
| 1000.0 | 1.133119005216772 |HER2mRNA
GEM
(ng/ml)
